# Epidemiology of keratitis/scleritis-related endophthalmitis in a university hospital in Thailand

**DOI:** 10.1038/s41598-021-90815-1

**Published:** 2021-05-27

**Authors:** Dhanach Dhirachaikulpanich, Kunravitch Soraprajum, Sutasinee Boonsopon, Warinyupa Pinitpuwadol, Preeyachan Lourthai, Noppakhun Punyayingyong, Nattaporn Tesavibul, Pitipol Choopong

**Affiliations:** 1 Department of Ophthalmology, Faculty of Medicine, Siriraj Hospital, Mahidol University, 2 Wanglang Road, Prannok, Bangkok-noi, Bangkok, 10700 Thailand; 2 Ophthalmology Services, Metta Pracharak Hospital, Nakhon Pathom, Thailand

**Keywords:** Diseases, Eye diseases, Infectious diseases

## Abstract

To demonstrate the demographics, associated factors, clinical presentations, microbiology, management, visual outcome and complications of keratitis/scleritis-related endophthalmitis (KSE). A retrospective chart of all endophthalmitis patients diagnosed between September 2001 and August 2011 was reviewed. Only endophthalmitis cases with previous corneal or scleral infection were included in the study. The patients were followed until losing vision or eyeball, becoming phthisis, or the end of 2018. Eighty-seven patients with KSE were identified, all unilateral. The mean age was 56.4 ± 21.4 years. There was a slight male predilection (55 patients, 63.2%). The mean follow-up time was 50 ± 149 weeks. The causative pathogens were identified in 35 patients (40.2%), with the highest frequency being bacteria. The most common bacterium was *Pseudomonas aeruginosa* (n = 13), and the most common fungus was *Aspergillus *sp. (n = 5). Fifteen patients achieved (17.2%) final visual acuity (VA) of hand motion or better after treatment. Eyeball removal was performed in 61 (70.1%) patients. From multivariate analysis, the only prognostic factor for poor final VA (worse than hand motion, HM) was poor VA (worse than HM) at the initial visit (relative risk 1.97, 95% confidence interval 1.15–3.36, p = 0.013). KSE is uncommon but has a devastating outcome. We found that the patient’s initial VA was the only predictor for their final vision. *P. aeruginosa* was the most common identifiable organism in this study. However, several fungal infections were recognised. These findings should raise awareness for treatment of KSE in the tropics.

## Introduction

Endophthalmitis, a severe infection inside the eye, is frequently caused by bacterial or fungal infection and leads to devastating sequelae. Endophthalmitis can be categorised into two types, exogenous and endogenous, depending on the way pathogens penetrate the eye. In exogenous endophthalmitis, pathogens directly invade the eyeball through a corneal or scleral wound, whereas in endogenous endophthalmitis, they enter through chorioretinal barrier via bloodstream. Keratitis/scleritis-related endophthalmitis (KSE), a subgroup of exogenous endophthalmitis, is caused by a severe ocular surface infection including ulcerative keratitis, stromal keratitis, and less commonly scleral infection. Ocular surface infection rarely develops into endophthalmitis, but when it does, the disease progresses aggressively and can lead to blinding consequences. To get rid of pathogen and prevent spreading of infection, most of KSE cases ended with evisceration or enucleation^1–10^. This disease resulted in much worse outcomes when compared with the other types of endophthalmitis^1^.

There were few studies of KSE worldwide; almost all were from temperate or subtropical regions. These reports demonstrated similarity in prognosis and causative pathogens. Patients mostly ended up with a poor final vision, and more than half underwent eyeball removal^2–4^. Bacteria, including *Staphylococcus* sp., *Streptococcus* sp., and *Pseudomonas aeruginosa*, are common causative pathogens for ocular surface infection leading to endophthalmitis^3,4^. However, a report from Florida demonstrated a higher rate of fungal infection than other reports from cooler and drier areas^2^.

From those studies, climatic differences may influence the type of causative agents for KSE. Nevertheless, there has been no study from tropical countries identifying pathogenic and visual prognosis variations. In this regard, the prevalence and outcome of KSE from tropical areas may represent the differences in pathogens and visual outcome. Unfortunately, there were only a few reports about endophthalmitis, and none of them mainly focused on KSE. The objective of this study is to show the demographics, associated factors, clinical presentations, microbiology, management, visual outcome and complications of KSE in a major university hospital in the central of Thailand over 10 years. The findings would represent epidemiology of KSE from the tropics.

## Material and methods

We conducted a retrospective chart review in accordance with the tenets of the Declaration of Helsinki and with the ethical guidelines for Medical and Health Research Involving Human Subjects. The study was approved by the Siriraj Institutional Review Board (SIRB), Siriraj Hospital, Bangkok, Thailand (Approval Number Si157/2012) and the need for informed consent was waived by the SIRB committee. The medical records of all endophthalmitis patients diagnosed between September 2001 and August 2011 at the Department of Ophthalmology, Faculty of Medicine, Siriraj Hospital, Mahidol University, Bangkok, Thailand were reviewed. We followed the patients until losing their vision to no light perception, getting eyeball removed, turning phthisis bulbi, or the study had reached the time limit at the end of 2018.

The definition of endophthalmitis included purulent intraocular inflammation as observed by slit lamp biomicroscopic examination or significant vitreous opacification as observed by ultrasonography. The treatments were mainly anti-infective agents. Patients with identifiable immunological or malignant causes were excluded. Only patients who were diagnosed previously with recent infectious keratitis or scleritis were selected for this study. The demographic data, associated factors, clinical manifestations at presentation, microbiologic profiles, management, and complications of the patients were obtained. The patients’ Snellen best-corrected visual acuity (BCVA) at initial presentation and the latest visit were collected. Counting fingers (CF), hand motions (HM), projection of light (PJ), perception of light (PL) and no perception of light (NPL) represented vision beyond Snellen range.

According to our institutional protocol, the conventional pathogen detection was performed originally and if necessary, the molecular method would be executed. The samples were inoculated onto blood and chocolate agars for bacterial culture, thioglycollate broth for anaerobic culture, non-nutrient agar with *Escherichia coli* for Acanthamoeba culture, and Sabouraud dextrose agar (SDA) with and without chloramphenicol for fungal culture in the routine laboratory. For aerobic bacteria, blood and chocolate agars were kept in a 5% CO_2_ incubator. For anaerobic bacteria, samples in thioglycollate broths were incubated in a gas-seal anaerobic jar along with H_2_ and CO_2_ generating system. Both aerobic and anaerobic cultures were incubated at 35 ± 2 °C with daily observation. Bacterial identification was made either by conventional technique or automated microbial identification system. For fungus, the SDA was incubated at 25–27 °C for 14 days, with daily observation. If hyphae or yeast were detected, the incubation period would be extended to 1 month, allowing colonization of slow-growing microorganisms. Mold identification was performed based on macroscopic and microscopic morphologies. The identification of yeast was based on phenotypic and morphological characteristics as well as biochemical approaches. The culture was reported as negative if no colony formed within 3 days for aerobic bacteria, 4 days for anaerobic bacteria and 14 days for fungus. To a less extent, direct PCR for the identification of bacterial and fungal infections was also applied.

Descriptive and analytic statistics were analysed using SPSS Statistics version 18.0 (SPSS, Inc., Chicago, IL, USA). Chi-square and Fisher's exact test were performed, and subsequently, multivariate analysis using generalised linear model (GLM) with log link function was applied to identify associated statistically significant factor for visual outcomes. The pre-specified confounders were age and sex. Relative risk (RR) with 95% confidence intervals (CI) was presented and P-value less than 0.05 (two-sided, 95% confidence interval) was considered as statistically significant.

### Ethics approval

The study was approved by the Siriraj Institutional Review Board. The informed consent was waived by the committee.

## Results

From 416 patients diagnosed with endophthalmitis in our institution, 87 patients with KSE (20.9%) were identified and reviewed in this study. The mean follow-up time was 50 ± 149 weeks. The longest follow-up case was 15 years. All cases were unilateral involvement with 41 (47.1%) having right eyes infected. The mean age of patients was 56.4 ± 21.4 years (median 60.2 years, range 2 months to 94 years). There were 55 (63.2%) males. Median time from onset of keratitis or scleritis to endophthalmitis was 9 days (interquartile range, IQR 4–26 days). Twenty-eight (32.6%) patients worked as construction workers, followed by 22 (25.6%) house workers and 11 (12.8%) agriculturists. Diabetes mellitus was the most common (16 patients, 18.4%) underlying systemic disease. Associated previous ocular abnormalities (including exposure keratitis, persistent epithelial defect, dry eye, glaucoma, previous ocular surgery, and diabetic retinopathy) were found in 31 (35.6%) patients. Corneal infection was the most common primary cause of KSE (70 patients, 80.5%). Table 1 demonstrated details of baseline characteristics and clinical features.

**Table 1:** Baseline characteristics of endophthalmitis associated with ocular surface infection in Siriraj hospital, Bangkok, Thailand from 2001 to 2011 (n = 87 patients, otherwise noted).

Characteristics	Pathogen unidentifiable (%)	Pathogen identifiable (%)	Total (%)
Mean age in years (SD)	56.4 (22.2)	56.3 (20.8)	56.4 (21.4)
Male	25 (62.5)	30 (63.8)	55 (63.2)
**Careers (n = 86)**			
Construction workers	14 (35.9)	14 (29.8)	28 (32.6)
Household workers	11 (28.2)	11 (23.4)	22 (25.6)
Agricultural workers	3 (7.7)	8 (17.0)	11 (12.8)
Others	11 (28.2)	14 (29.8)	25 (29.0)
Smoking history (n = 66)	9 (30.0)	9 (25.0)	18 (27.3)
Pre-existing ocular diseases	15 (37.5)	16 (34.0)	31 (35.6)
**Underlying diseases**			
Diabetes mellitus	8 (20.0)	8 (17.0)	16 (18.4)
Immunosuppressed	3 (7.5)	1 (2.1)	4 (4.6)
HIV infection	0	3 (6.4)	3 (3.5)
Malignancy	2 (5.0)	1 (2.1)	3 (3.5)
Chronic kidney diseases	0	2 (4.3)	2 (2.3)
Median time in days from onset of ocular surface infection to endophthalmitis (IQR)	8.5 (4–26)	10 (4–26)	9 (4–26)
Initial visual acuity less than hand movement	31 (77.5)	39 (83.0)	70 (80.5)
Right eye involved	19 (47.5)	22 (46.8)	41 (47.1)
**Locations of ocular surface infection**			
Cornea alone	31 (77.5)	39 (83.0)	70 (80.5)
Sclera alone	2 (5.0)	4 (8.5)	6 (6.9)
Corneoscleral	7 (17.5)	4 (8.5)	11 (12.6)
Ocular surface perforation (n = 72)	11 (33.3)	12 (30.8)	23 (31.9)
Hypopyon (n = 57)	17 (63.0)	19 (63.3)	36 (63.2)
Severe vitreous opacity (n = 80)	32 (88.9)	38 (86.4)	70 (87.5)
Prior treatment with antibiotics elsewhere (n = 76)	19 (59.4)	33 (75.0)	52 (68.4)

*SD* standard deviation, *HIV* human immunodeficiency virus, *IQR* interquartile range.

### Microbiology

Microbiology analysis was performed using various samples from conjunctival pus, corneal scrapes, aqueous fluid, vitreous fluid, or tissue biopsies. The highest yield was the culture from corneal scrapes (n = 16). Of note, we did not perform tissue biopsy and intraocular fluid analyses in every case. Microbiology data were missing in one patient, and we assumed the results as negative. Forty-seven cases revealed positive microbiology evidence as bacteria, fungi, or parasites. From these patients, four eyes demonstrated mixed pathogens (three with multiple bacterial infections and one with combined bacterial and fungal infections), whereas the others showed single isolation including bacteria (n = 25), fungus (n = 17) and Acanthamoeba (n = 1). Of those with positive results, we could further identify the genus or species of causative pathogens in 35 patients. The most common bacterium was *Pseudomonas aeruginosa* (n = 13), and the most common fungus was *Aspergillus* sp. (n = 5). Table 2 showed details of the causative pathogens in comparison with reports from different geographic locations.

**Table 2:** Identified causative pathogen species of the present study in comparison with other previous studies.

	Present study (n = 87)	Malihi et al.^4^ (n = 38)	O’Neill et al.^3^ (n = 37)	Henry et al.^2^ (n = 49)
**Geographic locations**	Bangkok, Thailand	New Jersey, USA	Melbourne, Australia	Florida, USA
**Bacteria, gram +**				
*Streptococcus *sp.	4	2	12	11
*Staphylococcus* sp.	2	10	8	1
Others	3	3	1	1
**Bacteria, gram −**				
*Pseudomonas aeruginosa*	13	4	11	8
Others	3	3	–	2
**Fungi**				
*Candida* sp.	–	–	–	5
*Aspergillus* sp.	5	–	–	1
*Fusarium* sp.	4	3	–	14
*Pythium* sp.	2	–	–	–
Others	2	1	–	6
**Parasites**				
*Acanthamoeba* sp.	1	–	–	–

### Treatment

All patients were admitted to our hospital for initial treatments. The median length of stay was 12 days (IQR 6–22 days). The initial broad-spectrum antimicrobials consisting vancomycin and ceftazidime with or without antifungal agents were administered through various routes until the microorganisms were identified. They were changed according to the laboratory results. Topical steroids were given in 8 patients as adjunctive treatments. In six of these patients, steroids were prescribed to prevent corneal graft failure. For the other two patients, they were prescribed later when the infection was controlled. Seventy-three (83.9%) patients required surgical treatments. Enucleation, the most common procedure, was performed in 61 patients (70.1%) (included one evisceration and one exenteration). Seven patients (8%) underwent penetrating keratoplasty, and three (3.4%) underwent pars plana vitrectomy. Median time from onset of endophthalmitis to date of first surgical treatment was 18 days (IQR 9–34.5 days). Fourteen eyes (16.1%) were treated without surgical interventions except for intravitreal antibiotic injections. The details of treatment according to pathogenic organisms were available in Supplement Table 1.

### Visual acuities and ocular complications

There were 17 (19.6%) patients with initial BCVA of HM or better. Only one patient had initial BCVA of 3/60, while the rest presented with beyond-Snellen visions ranged from counting fingers to NPL. After treatment, 15 (17.2%) patients achieved final BCVA of greater or equal to hand motions. Three (3.4%) patients recovered to functional vision (6/60 or better) (Table 3 and Supplement Table 1). The most common ocular complications or sequels included corneal scar (56, 64.4%), ocular hypertension (51, 58.6%), and panophthalmitis (16, 18.4%). The other complications included phthisis bulbi (9, 10.3%), iris neovascularization (3, 3.4%), cataract (3, 3.4%), and retinal detachment (2, 2.3%).

**Table 3:** Visual outcome of endophthalmitis associated with ocular surface infection in Siriraj hospital, Bangkok, Thailand.

BCVA	Initial BCVA, n (%)	Final BCVA, n (%)
6/60 and better	–	3 (3.4)
1/60—less than 6/60	1 (1.2)	1 (1.2)
Finger counts (FC)	1 (1.2)	1 (1.2)
Hand movement (HM)	15 (17.2)	10 (11.5)
Light projection (PJ) or light perception (PL)	42 (48.3)	6 (6.9)
No PL or eyeball removal	28 (32.2)	66 (75.9)

*BCVA* best corrected visual acuity.

### Prognostic factors

Logistic regression analysis was performed to identify the prognostic factors for the final visual outcome. From univariate analysis, the potential prognostic factors were identified, including initial BCVA, intravitreal antibiotics injection, and topical steroids use. Only poor initial VA indicated poor prognosis for final visual outcome in multivariate analysis (RR 1.97, 95% CI 1.15–3.36, p = 0.013) after adjusted for age, sex, intravitreal antibiotics injection, and topical steroids. The details of the analyses were shown in Table 4.

**Table 4:** Regression analyses indicated risk ratio of prognostic factors for poor final visual outcome (less than hand movement).

Prognostic factors	Crude risk ratio (95% confidence interval)	p-value	Adjusted risk ratio (95% confidence interval)	p-value
Age 60 years or more	1.10 (0.91, 1.34)	0.325	0.96 (0.88, 1.06)	0.421
Male	0.86 (0.72, 1.03)	0.105	0.99 (0.91, 1.07)	0.762
Presence of systemic diseases	0.90 (0.59, 1.36)	0.622		
Initial BCVA less than HM	2.42 (1.35, 4.34)	< 0.001	1.97 (1.15, 3.36)	0.013
Wound size > 5 mm	1.18 (0.89, 1.58)	0.207		
Ocular surface perforation	1.23 (1.04, 1.47)	0.088		
Hypopyon	1.21 (0.82, 1.79)	0.287		
Severe vitreous opacity	1.45 (0.87, 2.43)	0.052		
Pathogen identified	1.06 (0.87, 1.29)	0.530		
Bacterial infection	1.02 (0.80, 1.30)	0.888		
Prior antibiotics received elsewhere	1.15 (0.89, 1.49)	0.214		
Present to hospital later than 7 days after onset of endophthalmitis	1.23 (0.99, 1.53)	0.058		
Intraocular antibiotics treatment	0.73 (0.57, 0.93)	0.007	0.92 (0.81, 1.05)	0.22
Systemic antibiotics treatment	0.94 (0.71, 1.25)	0.99		
Steroids treatment	0.37 (0.16, 0.82)	< 0.001	0.52 (0.26, 1.06)	0.071

*BCVA* best corrected visual acuity, *HM* hand movement.

## Discussion

Endophthalmitis resulting from infectious keratitis/scleritis was uncommon; most studies often classified this aspect as the other cause of endophthalmitis. Here we reported the prevalence of KSE as high as 20% in our endophthalmitis cohort from Bangkok, Thailand. This result was more than twice the prevalence of KSE in previous reports worldwide^3,10^. In comparison with the result from the Northeastern part of Thailand, our proportion of KSE was much higher^11^. This finding probably reflected the etiologic differences from place to place even from the same country.

Our result showed that KSE caused a devastating visual outcome. Over 2/3 of our patients required eyeball removal while only 3 patients recovered to their functional vision. These findings were consistent with previous studies, in which patients mostly ended up with a poor final vision, and more than 50% underwent enucleation/evisceration^3,4^. Malihi et al. reported 38 infectious keratitides related endophthalmitis in New Jersey, USA^4^. Fifty per cent of cases were primarily enucleated. Similarly, of 37 cases gathered by the O'Neill group from Australia, more than 60% required evisceration or enucleation^3^. Another evidence was a study from Florida, USA, which reported 49 eyes of culture-proven microbial keratitis related endophthalmitis^2^. Although fewer patients underwent enucleation, about three quarters became legally blind. These results support the dire conditions of KSE.

*Pseudomonas aeruginosa* was found to be the most common pathogen identified in this study, which is similar to previous reports by Henry et al. and O'Neill et al.^2,3^. *Pseudomonas aeruginosa* uses various toxins to invade the cornea^12,13^. The toxins help the microbe to penetrate into the intraocular tissue through the intact cornea, allowing *Pseudomonas aeruginosa* to be the leading microorganism causing KSE worldwide^2,3,5,7,14–16^. According to its frequency and severity, initial antibiotics for keratitis should cover this organism, until proven otherwise, to prevent further progression of infection resulting in endophthalmitis.

Comparing isolated organism pattern, our study which was conducted in the tropical climate tends to have a high prevalence of fungal infection similar to the study from Florida where the climate is wet and warm^2^. Fusarium was identified as a frequent causative fungal pathogen as reported in the previous studies; however, *Aspergillus* sp. was not uncommon in our cohort^2,17^. This evidence emphasises the pattern difference of pathogenic organisms among climatic variations. Fungal infection should be considered more in patients living in a warm geographic location.

Differently from previous reports on KSE^2–4^, we identified two *Pythium insidiosum* and one *Acanthamoeba* keratitis that progressed to endophthalmitis among culture-positive cases. All three cases were enucleated and confirmed by pathological examinations. These organisms are common in wet tropical countries and can cause severe corneal ulcer. The progression of these infections to endophthalmitis was rare. However, when it did, the patients usually lost their eye. *Pythium insidiosum* is a fungal-like organism commonly found in tropical regions^18,19^. Water and wetland are the main reservoirs of this organism, making it a significant infection in Thailand. Due to its aggressiveness and antimicrobial resistance, most of the cases ended up with enucleation. Krajaejun et al. reviewed 102 nation-wide cases of human pythiosis in Thailand and reported 80% of ocular pythiosis required eyeball removal^19^.

Similarly, *Acanthamoeba* is an amoeba commonly found in tropical freshwater and swimming pools. Beside freshwater-related keratitis, it also frequently causes contact lens-related keratitis. Most patients received delayed treatment, and there is no specific antimicrobial agent. Patients with Acanthamoeba keratitis usually had a poor final vision. Previously, there were only a few case reports of acanthamoeba endophthalmitis associated with a history of contact lens use or immunocompromised state^20–23^. Although our case had no history of those and was primarily misdiagnosed as zoster keratitis, he had an agriculture-related work which could put him at risk for *Acanthamoeba* contamination. The confirmed diagnosis was obtained from corneal pathology after corneal transplantation. Eventually, our case required therapeutic enucleation similar to previous cases, signifying the severity of the disease.

Generally, pars plana vitrectomy is a favourite treatment option for endophthalmitis. This idea comes from the result of the Endophthalmitis Vitrectomy Study, which showed the benefit of vitrectomy in acute postoperative endophthalmitis patients with PL and worse^24^. However, in the case of KSE, the poor visualisation of intraocular structures due to corneal opacification may preclude the procedure and its benefits. As such, in our study, only 3 patients (3.45%) underwent vitrectomy. This finding was similar to a report by O'Neill et al.^3^. In contrast, enucleation was the primary treatment for our cases as it could urgently remove a bulk of causative organism and cease the progression. Therefore, vitrectomy may be indicated at an early stage of KSE when the corneal lesion was not extensive.

From univariate analyses, initial vision, intraocular antibiotics, and topical steroids indicated potential prognostic factors for final vision. However, these factors could confound the effect size from each other as initial vision might lead the ophthalmologist's treatment options. After adjustment, the poor initial VA was the only factor associated with the final vision worse than HM. Previous studies also indicated initial vision as an independent factor for visual outcome in other types of endophthalmitis^25–29^. In general, patients with poor initial VA suffered from severe infection or delayed treatment, making it more likely for them to undergo enucleation to get rid of infective agents and prevent further progression of the infection. Our result supported that initial VA was also a prognostic factor in KSE as these patients commonly presented with poor vision and ended up with enucleation or corneal scarring.

Apart from the initial VA, our univariate analyses denoted the use of intraocular antibiotics and adjunctive steroids as good prognostic factors. At present, intravitreal antibiotics injection is a standard primary treatment for endophthalmitis as supported by multiple studies^24,29–31^. Nonetheless, no randomised control trial studied about benefits of intravitreal antibiotics in KSE. In our cohort, we found its potential advantage in final vision; however, the number of cases was limited. In the previous case series, an intravitreal injection was administered between 50 and 100% of KSE contrasted to our study in which intravitreal antibiotics was injected in only 28.7% of cases^2–4^. This lower rate of intraocular antibiotics usage may happen due to the high rate of enucleation (70%) in our study. Nevertheless, our result showed the potential benefit of intraocular antibiotics for treatment of KSE in patients who do not need enucleation.

Although adjunctive steroids use showed potential in better final vision in univariate analysis, it did not indicate significant risk after adjusted for age, sex, initial vision, and intraocular antibiotics. This result may be according to the small number of cases and the selective bias in our study. We selectively use steroids in less severe cases when infection was under control. Anti-inflammatory effect of steroids may be helpful to prevent ocular structural destruction and preserve vision. On the other hand, it can simultaneously suppress host immunological responses, allowing invaded microorganisms to progress more actively. Topical steroids were reported to be associated with the progression of keratitis to endophthalmitis; nonetheless, a recent systematic review of four clinical trials was unable to conclude the risk or advantage of using adjunctive topical steroid in keratitis^2–5,17,32,33^. Moreover, another study also cannot conclude the benefit of adjunctive intravitreal dexamethasone in acute postoperative endophthalmitis^34^. Therefore, adjunctive steroids must be used with an awareness of the progression of the disease; concurrent aggressive antibiotics should be administered to control infection.

Interestingly, despite prior antibiotic treatment and the ability to identify causative pathogen, we did not find differences in visual outcome between those who had these factors or did not have them. These findings could be due to inadequate treatment and inability to early identify pathogens. KSE caused severe inflammation and destroyed both ocular surface and intraocular tissue leading to poor vision. Prior antibiotics treatment before the referral, which was usually commercially available fluoroquinolones or bacteriostatic antibiotics, was not sufficient to stop the infection. In addition, due to the scarcity and limited size of ocular specimens, causative pathogens usually be identified at the later course or unidentifiable. Consequently, the patients usually ended up undergone eyeball removal to prevent further damage of the surrounding tissue. Therefore, in practice, an early broad-spectrum antimicrobial and an attempt of pathogen identification are still recommended, however, the ophthalmologist should react quickly according to the patient’s clinical response and laboratory finding. In addition, understanding the prevalence of causative organisms in the local should support the antimicrobial selection for future patients.

The limitations of this study are the retrospective nature and a small sample size. Missing data prevented a full evaluation of important associated factors such as prior treatment of patients before referring to our hospital and initial clinical pictures prior to developing endophthalmitis. Furthermore, the lack of a specific protocol for the management and treatment of the patients may vary the outcomes. However, due to the rarity of cases, we tried our best to extract data to describe the epidemiologic picture of KSE from a large cohort of endophthalmitis in a tropical country like Thailand. In the future, a well-designed, multi-centred, prospective cohort study is recommended as it would provide a more accurate description of KSE.

## Conclusions

In conclusion, our study presented demographics, associated factors, and outcomes of KSE in a university hospital in Thailand. Although KSE is not common, this study highlighted its unfavourable outcomes in the tropical region. *Pseudomonas aeruginosa* was the most common identifiable organism in this study, although an ophthalmologist could not overlook fungal and other microbial infections. Only initial vision better or equal to hand motion was identified as a prognostic factor for good visual outcomes. More attention and further study are needed to find the prevention and management of this disease.

## Supplementary Information

Supplementary Table 1.

## Data Availability

All unidentifiable data are available at the corresponding author upon reasonable request.
