# Supplementary material for: Epidemiology of keratitis/scleritis-related endophthalmitis in a university hospital in Thailand
Source: Sci Rep. 2021 May 27;11:11217. doi: 10.1038/s41598-021-90815-1 (PMC8160326; doi:10.1038/s41598-021-90815-1)
Supplement: Supplementary file 1 — Supplementary Table 1. [file 41598_2021_90815_MOESM1_ESM.docx]

**Supplement Table 1** Treatment and visual outcome according to pathogen identification

|  | **Mixed pathogens**  **(n=4)** | **Single bacteria**  **(n=25)** | **Single fungi**  **(n=17)** | **Acanthamoeba**  **(n=1)** | **Pathogen unidentifiable**  **(n=40)** |
| --- | --- | --- | --- | --- | --- |
| Medication alone | 1 | 2 | 0 | 0 | 10 |
| Medication with surgery |  |  |  |  |  |
| - *Eyeball removal* | 3 | 17 | 14 | 1 | 26 |
| - *Penetrating keratoplasty* | 0 | 2 | 1 | 0 | 4 |
| - *Pars plana vitrectomy* | 0 | 2 | 1 | 0 | 0 |
| - *Others* | 0 | 2 | 1 | 0 | 0 |
| Visual outcome |  |  |  |  |  |
| - *Hand movement or better* | 0 | 5 | 2 | 0 | 8 |
| - *Less than Hand movement* | 4 | 20 | 15 | 1 | 32 |
